# Acoustic alarm signalling facilitates predator protection of treehoppers by mutualist ant bodyguards

**DOI:** 10.1098/rspb.2008.0410

**Published:** 2008-05-14

**Authors:** Manuel A Morales, Jennifer L Barone, Charles S Henry

**Affiliations:** 1 Department of Biology, Williams CollegeWilliamstown, MA 01267, USA; 2 Department of Ecology and Evolutionary Biology, University of ConnecticutStorrs, CT 06269, USA

**Keywords:** by-product mutualism, Formicidae, interspecific communication, Membracidae, substrate-borne signals

## Abstract

Mutualism is a net positive interaction that includes varying degrees of both costs and benefits. Because tension between the costs and benefits of mutualism can lead to evolutionary instability, identifying mechanisms that regulate investment between partners is critical to understanding the evolution and maintenance of mutualism. Recently, studies have highlighted the importance of interspecific signalling as one mechanism for regulating investment between mutualist partners. Here, we provide evidence for interspecific alarm signalling in an insect protection mutualism and we demonstrate a functional link between this acoustic signalling and efficacy of protection. The treehopper *Publilia concava* Say (Hemiptera: Membracidae) is an insect that provides ants with a carbohydrate-rich excretion called honeydew in return for protection from predators. Adults of this species produce distinct vibrational signals in the context of predator encounters. In laboratory trials, putative alarm signal production significantly increased following initial contact with ladybeetle predators (primarily *Harmonia axyridis* Pallas, Coleoptera: Coccinellidae), but not following initial contact with ants. In field trials, playback of a recorded treehopper alarm signal resulted in a significant increase in both ant activity and the probability of ladybeetle discovery by ants relative to both silence and treehopper courtship signal controls. Our results show that *P. concava* treehoppers produce alarm signals in response to predator threat and that this signalling can increase effectiveness of predator protection by ants.

## 1. Introduction

Mutualisms, defined as reciprocally beneficial interactions between species, are ubiquitous in nature despite early theoretical predictions of both ecological and evolutionary instability (Trivers 1971; May 1974; Axelrod & Hamilton 1981; Sachs & Simms 2006). Explaining this apparent paradox is one of the current goals underlying mutualism research (Hoeksema & Bruna 2000; Bergstrom *et al*. 2003). Evolutionary explanations for the stability of mutualism depend on whether these interactions are characterized by reciprocity, pseudoreciprocity or by-product benefits (Leimar & Connor 2003). These categories are distinguished by the extent to which benefits reflect partner investment (typified by reciprocity) or the side effect of behaviours that are independently adaptive for each partner (typified by by-product mutualism). Where benefit includes investment by one partner in exchange for by-product benefits from the other, the term pseudoreciprocity has been used (Leimar & Connor 2003).

Host–visitor mutualisms are consumer–resource interactions, in which one partner (the host) provides a resource reward in exchange for a visitor service (Thompson 1982). Ant-protection mutualisms are one important category of host–visitor mutualism that include both homopteran (=Auchenorrhyncha and Sternorrhyncha) and lepidopteran (=Lycaenidae and Riodinidae) hosts (Hölldobler & Wilson 1990). Although ecologically similar, ant–lepidopteran and ant–homopteran mutualisms differ fundamentally with respect to the nature of reward production. In lepidopteran hosts, ant rewards are produced specifically for ants by specialized organs, and this investment in reward production can be quite costly (Pierce *et al*. 1987). By contrast, for tended homopterans, ant rewards are fundamentally a waste product and investment in tending is often minimal (Stadler & Dixon 1999; Flatt & Weisser 2000; Morales 2000; Morales & Beal 2006). Because benefits in ant–homopteran mutualisms are closer to the case of by-product benefits while benefits in ant–lepidopteran mutualisms are closer to the case of pseudoreciprocity, they provide an ideal comparative system for understanding the evolution of mutualism.

Interspecific signalling is increasingly recognized as an important mechanism underlying the regulation and coordination of investment between mutualist partners. It can thus play a critical, albeit understudied (Kostan 2002), role in the evolution of these interactions (Noë & Hammerstein 1994; Leimar 1997). Among insects, substrate-borne vibrational signalling is a widespread mode of communication (Wilson 1971; Lewis 1984) and it plays a role in behaviours ranging from courtship and mating to predator defence to conveying the location of food resources (Henry 1985; Cocroft 2001). At least two studies have documented substrate-borne signalling between partners in the mutualism between ants and lepidopteran caterpillars (DeVries 1990; Travassos & Pierce 2000). These previous studies conclusively demonstrate that interspecific signalling by lepidopterans can play an important role in regulating investment levels by ant mutualists.

While there is a substantial literature documenting the diversity and function of interspecific signalling in ant–lepidopteran mutualisms (Leimar & Axén 1993; Axén *et al*. 1996; Fiedler *et al*. 1996; Agrawal & Fordyce 2000; Travassos & Pierce 2000), few studies have addressed signalling in ant–homopteran mutualisms, and none have focused on acoustic signalling between mutualists (Cocroft 1996). In this paper, we consider the possible role of vibrational signalling for the ant-tended treehopper, *Publilia concava*. In §4, we place these results in the context of previous work on signalling in ant–lepidopteran mutualisms, and consider the implications of this contrast for understanding the evolution of mutualism and for signalling in these systems.

## 2. Material and methods

### (a) Encounter trials

To test whether signals were produced in a defensive context, we placed a single ladybeetle that had been starved for 24–48 hours onto a potted goldenrod plant with an untended adult female treehopper and her brood. Ladybeetles will feed on treehopper nymphs (juveniles) that are defended by adult treehopper females (see S1 and S2 in the electronic supplementary material). For any trial in which the predator encountered the treehopper (defined as physical contact between the two), we determined the number of signals produced during the 10 s interval immediately preceding the encounter and the 10 s interval starting at the time of contact. We chose a 10 s interval for analysis because this captured the minimum period of initial contact between treehoppers and ladybug beetles. Nevertheless, we note that repeated bouts of contact were commonly observed—at least one-third of encounters lasted for more than 20 min (most trials were ended before signalling stopped). We evaluated a total of 10 unique treehopper–predator encounters (10 treehopper individuals and 10 predator individuals). In most cases, the ladybeetle did not encounter the adult during the first trial; these adults were retested until they were encountered by a ladybeetle. To determine whether signals were produced when encountering ants, the same procedure was used but replacing beetle predators with ants (*n*=10 ants and 10 treehoppers). Insects used in these experiments were from sites located in Williamstown, MA, USA. The same treehoppers were used in predator and ant encounter trials with a minimum interval of 3 days between trials and with the sequence of encounters randomly assigned (treehopper identity was included as a random effect in analyses, see below). Thus, in these experiments the appropriate comparison includes both a temporal (before and after contact) and treatment (predator versus mutualist) contrast.

### (b) Signal recording and analysis

We recorded all vibrational signals during the encounter trials using an ICP accelerometer connected to a battery-powered signal conditioner (352C65 and 480E09, PCB Piezotronics, Inc., Depew, NY) at a voltage gain of 10. The accelerometer (2.26 g, approx. 11×7.5×7.5 mm) was attached to the plant using beeswax. We digitally recorded signals at a bit rate of 16 and a sampling rate of 48 kHz using either a digital audio tape deck (TASCAM DA-P1, TEAC America, Inc., Montebello, CA) or a DVCAM recorder (Sony DSR-PD100, Sony Electronics, San Diego, CA).

Using a mixed-effects model, 84 signals from the 10 s interval immediately following predator contact were analysed (maximum of 10 signals for each of eight treehoppers; two treehoppers were excluded due to poor quality recordings). This analysis allowed us to partition the variance components between and within individual treehoppers (Pinheiro & Bates 2000). We examined the following four properties of signals: (i) peak frequency was calculated from the smoothed spectrum as the frequency with maximum power, (ii) bandwidth was calculated from the half-power points—the frequencies at which the power had decreased by 3 dB relative to the peak frequency, (iii) duration of each signal was measured from the waveform by visually identifying the beginning and end of each pulse with respect to the background noise level, and (iv) pulse rate of signals was calculated as the inverse of the time between the beginning of one signal and the beginning of the next.

### (c) Predator discovery trials

To test the effect of signal production on the probability of predator discovery by ants, we staged predator ‘attacks’ in a series of 10 min field trials during the summer of 2006. For experimental trials, we reproduced a previously recorded beetle–treehopper encounter (see S3 in the electronic supplementary material) using an electrodynamic shaker (ET-132-203, Labworks, Inc., Costa Mesa, CA) attached approximately 6 cm above the uppermost nymph aggregation (interquartile range=5.0–6.5 cm; range=2.5–22 cm). Note that the distance from shaker to aggregation had no effect on the probability of discovery and was not included as an explanatory variable in analyses. Control trials were handled identically, but no signal was played. Trials began 5 min after positioning the shaker by placing a ladybeetle on the leaf nearest to the point of shaker attachment and beginning playback for the signalling trials. Trials ended when ants contacted ladybeetles or until 10 min following ladybeetle introduction in the absence of contact. We selected only aggregations (defined as all treehoppers on a given plant) guarded by at least one female and tended by at least two ants. We conducted a total of 64 trials on 34 plants using a paired design where possible and with each plant separated by at least 5 m. The order of signal presentation was randomly determined. Paired trials were separated by a minimum of 1 day. When treehopper females abandoned their brood in the interval between rounds, a new plant was selected (4 out of 64). The number and species identity of ants was recorded at the beginning of trials.

The amplitude and power characteristics of reproduced signals as recorded directly opposite the accelerometer closely matched that of the originally recorded signal. We detected no bias in the peak frequency of the reproduced signal as a function of distance from signal injection compared with the original signal, and visual inspection of the spectrum showed no systematic bias in the frequencies.

To test the hypothesis that alarm signal production rather than signalling *per se* increases the probability of ladybeetle discovery by ants, we repeated the experiment in 2007 adding a second control consisting of a courtship signal recorded from a male *P. concava* treehopper (see S4 in the electronic supplementary material). We conducted a total of 78 trials on 45 plants, again using a paired design where possible. In addition, we collected data on the number of patrolling ants at the end of each trial.

### (d) Statistical analysis

Because signal production is a discrete response with relatively low frequency of occurrence (i.e. non-normal), and to address the non-independence of trials resulting from using a paired design, we analysed treehopper encounter experiments using a mixed-effects generalized linear model with Poisson errors and treehopper as the grouping variable (Faraway 2006; Bates & Sarkar 2007). Similar methods were used for analysis of the ant-activity data and playback trials but including initial ant abundance as a covariate and, for the analysis of playback trials, binomial errors.

For mixed-effects models, likelihood-ratio tests of the fixed effects are anti-conservative (Pinheiro & Bates 2000) and for generalized mixed-effects models, both the distribution and the denominator degrees of freedom for *F*-tests are based on untested approximations (Littell *et al*. 1996). Consequently, we evaluated the significance of fixed effects using numerical methods (Markov chain Monte Carlo sampling) to sample the empirical distribution of parameter values. One-tailed *p* values were used to test the specific hypothesis that alarm signal playback increased the probability of ladybeetle discovery relative to controls, calculated as the fraction of samples overlapping zero. Two-tailed *p* values were used for all other analyses, calculated as the fraction of residuals whose absolute value was greater than the absolute value of the mean. All statistical analyses were conducted in the statistical environment, R (R Development Core Team 2005).

## 3. Results

**Figure 1:**
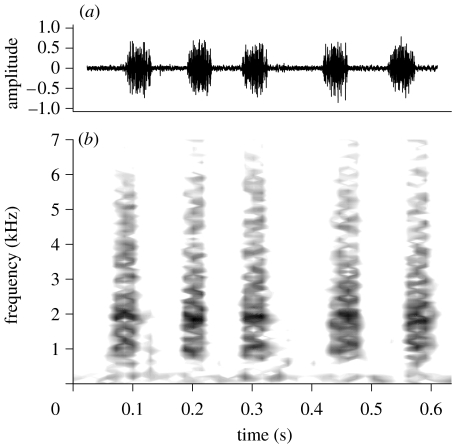
(*a*) Spectrogram and (*b*) waveform of the vibrational signal produced by the treehopper *P. concava* in response to disturbance.

**Figure 2:**
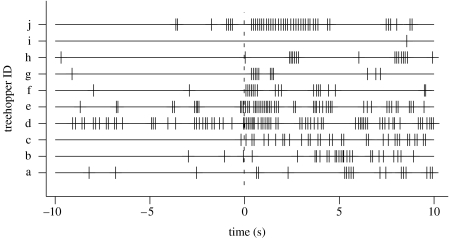
The temporal pattern of alarm signal production recorded during predator-encounter trials. In each trial, the timing of predator contact is indicated by the dashed vertical line at time zero.

*Publilia concava* adults of both sexes produce a low-amplitude substrate-borne vibrational signal when disturbed (figure 1; table 1; see S3 in the electronic supplementary material). Signals were typically produced in ‘volleys’ of at least three units, although we occasionally observed them produced singly (figure 2).

**Figure 3:**
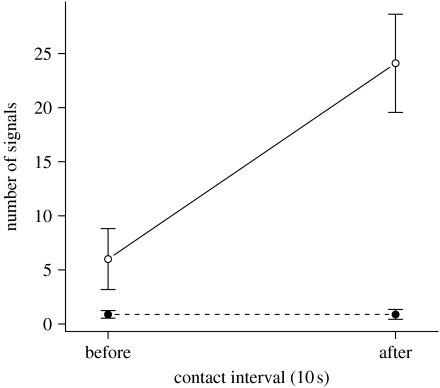
Mean (±s.e.) signals produced by *P. concava* treehopper females in the 10 s prior to and following contact with a predator (open circles) or ant (filled circles).

In experimentally staged encounter trials, alarm signal production increased by a factor of four following contact with predators (see S1 and S2 in the electronic supplementary material) but remained unchanged following contact with ants (figures 2 and 3, table 2). Notably, the small but significant increase in signalling prior to contact in ladybeetle trials (table 2) indicates that treehoppers are capable of identifying ladybeetles even prior to initial contact.

**Figure 4:**
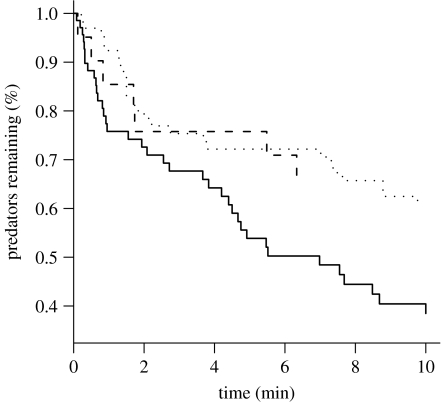
The probability that a ladybeetle will remain undiscovered by ants as a function of time (min) and playback treatment. Solid line, alarm; dashed line, courtship; dotted line, control.

To determine the functional significance of alarm signalling for predator protection by ants, we evaluated the probability of predator discovery by ants during signal playback and control conditions. Because predator discovery did not vary with either year or ant species (year+spp.|trt+no. of ants; *Χ*_4_^2^=3.36, *p*=0.5; 92% of aggregations were tended by species in the *Formica* ‘fusca’ group), data were pooled across years and species was excluded from the analysis. Predator discovery by ants was significantly enhanced by playback of alarm signals relative to both silence and courtship signal controls—the odds of beetle discovery increased by a factor of 2.7 and 2.9, respectively, during alarm signal playback (figure 4; table 3). There was no difference in the probability of ladybeetle discovery between silence and courtship treatments (figure 4; table 3).

**Figure 5:**
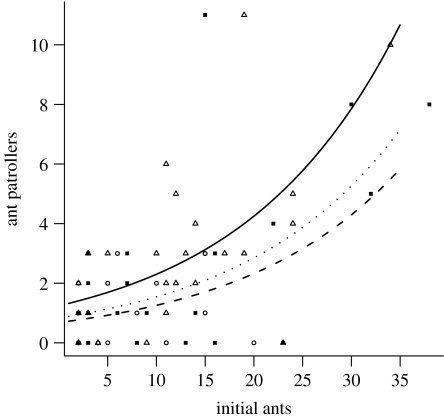
The number of patrolling ants at the end of playback trials relative to the number of ants at the start of trials for alarm signal, courtship signal and silence treatments. Triangles, alarm; circles, courtship; squares, control.

Notably, the probability of beetle discovery in playback trials was mirrored by changes in ant activity measured at the end of trials (figure 5; table 4). Playback of alarm signals increased the total number of ants patrolling plants relative to both silence and courtship signal controls by a factor of 1.4 and 1.9, respectively (figure 5; table 4). There was no difference in ant activity between silence and courtship signal treatments (figure 5; table 4).

## 4. Discussion

Our results show that treehoppers signal in response to predator threat, and that this signalling increases both ant activity and the probability of predator detection by ants. We did not observe signalling in response to contact with ants, suggesting that signalling is fairly specific to instances of predator attack in this system. Additionally, we did not observe an increase in ant activity or predator discovery following playback of a male courtship signal, suggesting that the response of ants to this alarm signal is not a general response to any vibrational signal.

In contrast to the predator-specific signalling that we observed, acoustic signalling in ant–lepidopteran mutualisms occurs more or less continuously and responds to both ant presence and simulated threat of predation (DeVries 1990; Travassos & Pierce 2000). One explanation for the relatively low frequency of signalling in this study is that signalling represents honest communication in *P. concava* (alerting ants to predator presence) but includes a component of dishonest communication in lepidopterans (serving to regulate their investment in ant tending). For example, lycaenids may ‘train’ ants to respond to signalling using an intermittent reinforcement strategy, thereby reducing the cost of reward production. Indeed, a number of studies suggest that lepidopterans use a variety of chemical and behavioural strategies to fine-tune levels of investment in ant tending (DeVries 1988; Leimar & Axén 1993; Axén *et al*. 1996; Fiedler *et al*. 1996; Agrawal & Fordyce 2000).

Ultimately, differences in the frequency of signalling between these taxa may follow from differences in the relative cost of reward production. If the energetic cost of signal production is lower than the cost of producing ant rewards, signal production could represent a ‘lower-cost strategy’ for attracting ants. The high efficiency of energy transfer for vibrational signals (Virant-Doberlet & Cokl 2004) is consistent with the hypothesis that signalling can represent a lower-cost strategy for attraction. Moreover, the fact that homopterans provide ants with an excretion that must be produced regardless of ant presence whereas lepidopterans provide ants with a secretion specifically produced for ants suggests that the cost of reward production is substantially higher for lepidopterans (Leimar & Connor 2003).

Perceived differences in the cost of reward production between these taxa have led to the classification of ant–lepidopteran mutualisms as examples of pseudoreciprocity but to classification of ant–homopteran interactions as examples of by-product mutualism (Leimar & Connor 2003). Notably, signalling in by-product mutualisms is predicted to increase coordination of benefits (i.e. the efficiency of interaction; Leimar & Connor 2003), whereas signalling in mutualisms characterized by some degree of reciprocity is predicted to mediate the level of investment among partners (e.g. by restricting reward production to instances of greatest need; Leimar 1997; Leimar & Connor 2003).

Although few studies are available that have evaluated both patterns of signalling and costs of reward production, relevant data are available for the treehopper used in this study, *P. concava*, and the lycaenid *Jalmenus evagoras*. *Publilia concava* treehoppers are significantly larger when tended by ants (Morales & Beal 2006) suggesting a low cost of ant tending, whereas larvae and pupae of the lycaenid *J. evagoras* are significantly smaller when tended by ants, suggesting a substantial cost of ant tending (Pierce *et al*. 1987). This high cost of ant tending in *J. evagoras* is associated with a high frequency of ant-dependent signalling, in contrast to the results for *P. concava* presented here. Combined, these observations support the hypothesis that lepidopterans use acoustic signalling to minimize investment in ant tending by communicating partner quality (i.e. needs and abilities; Axén *et al*. 1996; Leimar 1997; Agrawal & Fordyce 2000), whereas signalling by *P. concava* treehoppers functions primarily as an alarm signal thus increasing coordination of benefits with ants.

### (a) Alternate hypotheses and caveats

We show an increase in ant activity and a corresponding decrease in the time to predator discovery by ants following signal playback, although we note that other non-exclusive hypotheses may explain signalling by treehoppers in response to predators. In particular, signalling may serve to warn other treehopper nymphs or adults, or act to repel predators. Indeed, alarm signalling in the context of maternal defence from wasp predation has been previously demonstrated for the untended treehopper species *Umbonia crassicornis* (Hemiptera: Membracidae). Nymphs of this species produce a coordinated group alarm signal in response to predator attack that elicits anti-predator behaviour on the part of the parent female (Cocroft 1996).

Although the hypotheses outlined above are not addressed by our study, previous studies on the role of maternal care in *Publilia* spp. treehoppers suggest that these alternate hypotheses are less likely. Because the benefit of maternal guarding depends on the presence of ants (McEvoy 1979; Billick *et al*. 2001), signalling is unlikely to directly influence predator success. Rather, previous studies of maternal care in *Publilia* spp. have suggested that mothers primarily benefit nymphs by maintaining a standing guard of ants or by increasing ant-tending levels. Our results suggest a specific mechanism—interspecific acoustic signalling—that may enhance this effect.

A substantial body of work has addressed the potential problem of generalizing conclusions from analysis of a single signal to a whole class of signals (Gerhardt 1992). More generally, this issue is known as external validity and is relevant when extending any conclusion beyond the range of treatments, environmental conditions, spatial location and even individuals used in a given study (Gerhardt 1992). In this study, our predator-discovery trials used a single exemplar for the playback signals and therefore our conclusions are strictly limited to the particular signal used in these experiments. However, as others have argued, the generality of conclusions ultimately needs to be based on biological plausibility (Gerhardt 1992). We believe that our results are generally applicable for several reasons. First, an analysis of the variation between randomly sampled 10 s intervals of the playback signal matches the pattern of variation between treehoppers for three of the four signal characteristics measured, especially in comparison with the variation within treehoppers (table 1, see S6 in the electronic supplementary material). Second, laboratory trials of ant foraging response to artificial nectaries using a different synthesized playback signal showed a shift in foraging behaviour from feeding to patrolling (J. L. Barone & M. A. Morales 2003, unpublished data), consistent with the results of the current study. Finally, although our results differ in detail, the pattern of ant response to treehopper signalling is largely consistent with the results from ant–lycaenid studies showing ant responses to similar broadband signals (DeVries 1990; Travassos & Pierce 2000). Future work exploring how variation in signal properties reflects differences in ant behaviour will provide valuable insight into the evolution of signalling in this system.

## 5. Conclusions

Our study joins an increasing number (DeVries 1990; Travassos & Pierce 2000) suggesting that vibrational communication may be widespread in ant associations. Nevertheless, we document substantial differences in the frequency and ant-dependent context of interspecific signalling for this ant–treehopper mutualism (alarm signalling), relative to previous studies of ant-tended lepidopterans (recruitment signalling). We suggest that the evolutionary trajectories of interspecific signalling in these two ant-protection mutualisms have been partially shaped by differences in the nature of reward production and concomitant costs of association. Future studies across a range of taxa should provide important details on the evolution of character traits within these ecologically similar mutualisms, and more generally should provide important insight into the evolution of mutualism itself.

## Figures and Tables

**Table 1:** Frequency and temporal properties of treehopper alarm signals compared within and between individuals (*n*=82 signals, 10 treehoppers).

	mean	s.d._within_	s.d._between_
peak frequency (Hz)	1722	179	403
bandwidth (Hz)	1112	295	133
signals (s)	8.2	3.8	2.5
signal duration (ms)	43.9	5.6	2.8

**Table 2:** Poisson regression analysis of treehopper signalling (number of signals per 10 s interval) following ant and predator encounters with treehopper as a grouping variable (i.e. random effect).

model coefficient	exp(estimate)^a^	95% CI	*p* values
predator versus ant encounter	6.73	0.80 to 3.23	0.003
time	0.99	−1.01 to 1.01	0.994
treatment×time	4.04	0.35 to 2.45	0.01

a Relative change.

**Table 3:** Logistic regression analysis of ladybeetle discovery as a function of signal playback and ant-tending level with plant as a grouping variable (i.e. random effect). (Note that contrasts are presented to highlight the effect of alarm signal playback relative to controls.)

model coefficient	exp(estimate)^a^	95% CI	*p* values
alarm signal versus silence control	2.72	0.19 to 1.76	0.008^b^
alarm signal versus courtship signal	2.99	−0.02 to 2.19	0.028^b^
courtship signal versus silence control	0.86	−1.21 to 0.99	0.847
number of ants	1.03	0.01 to 0.05	<0.001

a Odds ratio.

b One tailed (see §2).

**Table 4:** Poisson regression analysis of ant activity following playback trials with plant as a grouping variable (i.e. random effect). (Note that contrasts are presented to highlight the effect of alarm signal playback relative to controls.)

	exp(estimate)^a^	95% CI	*p* values
alarm signal versus silence control	1.43	0.05 to 0.75	0.005^b^
alarm signal versus courtship signal	1.9	0.15 to 1.08	0.012^b^
courtship signal versus silence control	0.75	−0.73 to 0.27	0.388
number of ants	1.06	0.05 to 0.08	<0.001

a Relative change.

b One tailed (see §2).

## Supplementary Material

S1 Video showing a treehopper–ladybeetle encounter (MPEG-4 MOV; 3.1 MB)

S2 Video showing a treehopper–ladybeetle encounter. Note wing-flicking defensive behaviour by treehopper at approximately 0.11 and 0.14 s (MPEG-4 MOV; 3.1 MB)

S3 Audio recording of the treehopper–ladybeetle encounter used in playback trials. The full encounter lasted >20 min—the first 10 min was used for the playback trials (in which the original uncompressed file was used). (MP3; 4.6 MB)

S4 Spectrogram of a male treehopper courtship signal. (PDF; 372 kB)

S5 Audio clip of male treehopper courtship signal used in playback trials (looped for 10 min using the original uncompressed file)

S6 Frequency and temporal properties of the alarm signal used in playback trials compared within and between 10 second intervals (N=173 signals, 10 intervals).
