# Supplementary material for: Acoustic alarm signalling facilitates predator protection of treehoppers by mutualist ant bodyguards
Source: Proc Biol Sci. 2008 May 14;275(1645):1935–41. doi: 10.1098/rspb.2008.0410 (PMC2627810; doi:10.1098/rspb.2008.0410)
Supplement: S6 — Frequency and temporal properties of the alarm signal used in playback trials compared within and between 10 second intervals (N=173 signals, 10 intervals). [file rspb20080410s20.html]

**S6.** Frequency and temporal properties of the alarm signal used
in playback trials compared within and between 10 second intervals (N
= 173 signals, 10 intervals).

|  | **Mean** | **SDWithin** | **SDBetween** |
| --- | --- | --- | --- |
| Peak Frequency (Hz) | 1927 | 81 | 30 |
| Bandwidth (Hz) | 996 | 241 | 68 |
| Signals/s | 4.0 | 2.8 | 2.1 |
| Signal duration (msec) | 49.6 | 5.3 | 1.7 |
